# Remarks, Gleanings, and Extracts

**Published:** 1874-03

**Authors:** 


### REMARKS, GLEANINGS AND EXTR&CTS

BY LOCAL EDITORS.

New Views on Diabetes.—M. Lecorche has submitted to the Academy of Medicine of Paris, the following opinions respecting the nature of diabetes:—1. The current theories touching the pathology of diabetes refers only to certain varieties of glycosuria which have nothing to do with diabetes. They do not explain diabetic glycosuria. 2. Glycosuria, in diabetes, is only a secondary circumstance; the principal phenomenon is a tendency to disassimilation of protein substances. Diabetes may, in fact, be called azoturia. This disassimilation is the very essence of diabetes, and is characterized by the enormous quantity of urea which the patient is daily losing. 3. This protein disassimilation is the primary cause of glycosuria, which latter is simply an unimportant sequal of that cause. Protein disassimilation requires combustion, and during this combustion the oxygen leaves unattacked any glycosic substance formed in the economy; hence the existence in the urine of a quantity of sugar, which quantity increases with the amount of urea. 4. These views of the pathology of diabetes are of capital importance as regards the treatment, for they pave the way to a rational mode of treating the disease. The theories hitherto offered do not admit of such a course, as they refer only to glycosuria.

In viewing diabetes as M. Lecorche proposes (i. e., as azoturia, of which the glycosuria is the consequence) there is, he says, only one way of contending with the disease—namely, to endeavor, by every means in our power, to stop the loss of urea experienced by the patient. To attain this end we have only one mode of treatment at our command—the administration of cumulative remedies. Among these the principal are opium, arsenic, valerian, and perhaps bromide of potassium.

M. Lecorche promulgated these opinions before the Academy at the meeting of June 10th last, and promises to give further developments (and it is to be hoped experimental proofs) in the publication of lectures on diabetes delivered by him at the Faculty.—*Lancet.*

Chloral Hydrate in Incontinence of Urine.—In the *London Lancet* for December, Dr. William Thomson attests the virtue of chloral hydrate in incontinence of urine, when difficulty is the result of habit. In fifteen cases it had worked a perfect cure within a week. He lays down the following rules for its exhibition:

1. Give it only at night and in bed, and after two hours of fasting.
2. Give it in full doses.
3. Use but little drink, and no intoxicating liquor.
4. If it should fail, do not continue it beyond a week or ten days.

According to our own experience, belladonna is quite as effectual. To a child of three of four years, give 10 drops of the tincture on going to bed. The dose may be slowly increased, should it fail at first, until the pupils dilate. If, at this stage, the incontinence continue, no benefit is to be expected from longer use.—*Pacific Medical Journal.*

Anæsthetics in Midwifery.—Dr. Dougan Clarke, in the *American Journal of Medical Science*, publishes an interesting paper from which we find in the *Medical Archieves*, the following important statements:

"1. In perfectly nominal labors of moderate suffering and duration and without untoward symptoms of any kind, anæsthetics should be resorted to.

"2. If the pains, even in an otherwise normal labor, be inordinate and protracted, especially in the second stage, anæsthetics are indicated.

"3. Irregular pains, exhaustive in their character, and inefficient, may be tranquilized by anæsthesia, and afterward the action of the uterus is, as a rule, more regular and efficient.

"4. Threatened convulsions may be averted, and those existing may be terminated, by a judicious use of anæsthetics. In cases of eclampsia, however, with congestion of the brain, or organic disease of that organ from any source, they are contra-indicated.

"5. Anæsthesia is proper in all obstetrical operations, whether manual or instrumental. It is useful in eversion, embryotomy, and the use of the forceps, and indispensable in Cæsarean action.

"6. Anæsthetics are among the most reliable means of overcoming rigidity of the perineum, os uteri, or vagina, especially when depending upon muscular excitement.

"7. Chloroform or ether may be employed for anæsthesia, of which chloroform is the most convenient, agreeable and efficient, but ether in midwifery, as in surgery, is the most safe.

"8. Chloroform is much less dangerous in midwifery than in surgery, so far as past experience can determine.

"9. It is not necessary, as a rule, to bring about complete insensibility, but only sufficient to obtund the severity of the pains. The exceptions to this rule will readily suggest themselves to a thinking mind."

Clark's Powders for Intermittent Fever.—℞. Quinæ sulphate, grs. x.; pulv capsici. grs. iij.; pulv. opii, grs, .i. M

The patient receives one powder twelve hours previous to the time of the chill, and another about an hour previous. The powders are continued in this manner for three or four days, and then continued every morning as long as may be desired. The most usual time for the chills to return is at the end of two weeks, when it is necessary to reinforce the system by the full sized powders for three or four days, (if they have been diminished,) and to be given twice a day.

The certain arrest of the disease is expected under this treatment. Iron should be administered after the chills are stopped.—*Medical Record.*

STRYCHNIA WITH ATROPIA IN OPHTHALMIA.—Dr. T. Barrows (*Medical and Surgical Reporter,*) says: Prof. George B. Wood, M. D., in U. S. Dispensatory, No. 12, page 564, line 23 from bottom, says that the action of nux vomica is directed more particularly to the paralytic part. He also affirms that the effects of strychnia are precisely similar. In a solution of strychnia gr. ij.; with atrophia, gr. ⅛, the expansive effects of the atrophia are vastly greater than the contractile effects of the strychnia.

Having myself very often suffered from ophthalmia, occasioned by previous long continued and excessive use of artificial light, and having obtained only partial relief after several days' trial of such remedies as plumbi acetat with tinct. opii dil., alum et potas. sulph., cupri sulph., cold water, etc., I at length, encouraged by the above mentioned statement of Dr. George B. Wood, prescribed for myself. ℞. Atropiæ, grs. j⅛.; strychniæ, grs. ij.; acid acetat, gtt. j. or q. s., aquæ rosar, fl℥ iv. M. Dose one drop in each eye once daily. This combination acted like a charm, usually rendering the eyes clear in a few hours. For weakness of my eyes I have used: ℞. Strychniæ, grs. ij.; acid acetat, gtt. vj, or q. s.; aquæ rosæ, fl℥ vj. Dose 1 drop in each eye thrice daily. I have continued this practice once, twice or thrice daily, for I think about 17 months; during which I have suffered only once, and but slightly from ophthalmia, occasioned by coryza, about two weeks since. This is a longer exemption than I had enjoyed during the previous 14 years. For dullness of hearing I have employed: ℞ Strychniæ, grs. viij.; acid acetat, gtt. v., or q.s.; aquæ rosar, fl℥ ij. M. Dose 1 drop in each ear once a day. My dullness of hearing has by this means been much diminished; as has also that of Mrs. Brooks; a friend of Mrs. Brooks', Mr. Turner and his father-in-law, and several others. For acute ophthalmia I usually prescribe: ℞. Atropiæ, grs. ⅛.; strychniæ, grs. j.; aquæ rosæ, fl℥ j. M. Put 1 drop in each eye once a day. Many have been promptly benefitted by means of this combination.

Miss Martha Robinson had suffered severely from ophthalmia with conjunctivitis throughout two years. The treatment she received during 18 months produced very little visible relief. I prescribed for her the first of the preceding formulæ, together with ung. hyd. rubri. Within two weeks her eyes and eyelids were completely relieved. One or two slight relapses have promptly yielded to the same remedies. I saw her a week ago, and was pleased to see her eyes looking bright and clear.

Miss Agnes Whitlock had suffered severely from ophthalmia with conjunctivitis, throughout 5 years. She was obliged to abandon school on account of it; and although she habitually wore a shade, and occupied a darkened room, she complained bitterly of the light hurting her eyes when she came in to attend meals. The treatment to which she submitted during this time produced only slight and transient amendment.

I prescribed for her the first of the preceding formulæ, with ung. hydrarg. rub. Within two weeks her eyes became normal, and with the aid of the same ℞. and strychnia, which I have previously stated I use myself, she has had no relapse at all during about 19 months.

I hope that the preceding facts and views may lead some physician to use either the preceding formulæ, either as they are, or somewhat modified, in treating ophthalmia, week eyes, and partial deafness.

As coryza will be somewhat prevelent till about next April, please allow me to add my formula for it to the several which have already been furnished to your excellent journal. ℞. Pulv. cubeb, ʒij.; pulv. cupri sulph., grs. ij. M. In one box. Snuff up a small pinch about every two hours till relieved.

Frontal Catarrh.—Dr. J. S. Prout, of Brooklin, N. Y., in an article on frontal catarrh, published in the *Medical Record*, said, let me, then, without further introduction, state that I have, in my own person, and with patients, often been able to arrest the disease in the course of an hour or less, by taking or giving large doses of the officinal tincture of chloride of iron, 20 or 30 minims, as soon as possible after the cold is "caught." I generally find that in about half an hour there is a decided amelioration of symptoms, which may be permanent, in which case I take or give more of the tincture, or the improvement may pass off in two or three hours; in which case, the dose must be repeated. This may be required three or four times.

I have had numerous attacks of frontal catarrh, which I have thus caused to abort. I have had the same good fortune with coryzas accompanied by sore throat.

In other cases, perhaps on account of greater severity or from delay in commencing the treatment, I have not obtained permanent benefit from the use of the tincture. In my hands, therefore, it is not a specific, though I have great confidence in its efficacy.

A convenient form for extemporaneous prescription is—℞ Tinct. ferri chloridi, glycerine, aa., ʒiv. M. S. One teaspoonful in a wine glassful of cold water, through a glass tube, to be repeated according to circumstances. The glycerine in part conceals the iron taste.

A Good Laxative.—℞. fluid ext. sennæ; tinc. rhei aa. 1 oz.; fluid ext. leptandra, ½ oz. M. Dose one teaspoonful.

CAPPILLARY BRONCHITIS—TREATMENT OF.—1. Free diaphoresis, warm room, warm coverings, warm drinks, hot foot baths.

2. Warm or hot applications to the chest (Niemeyer even gives *hot* baths in extreme cases; mustard poultices, hot flannels, etc., are used as hot as can be borne.

3. For cough, morphia, atrophia, hydrocyanic acid, chloroform, ether, etc. I greatly prefer bromide of potassium and chloral, used very gradually.

4. Small doses of ipecac., tartar emetic, etc., in early stages.

5. If the secretions be tough, the alkalies and chlorides.

6. Tonics as soon as fever subsides; blisters, if required; painting with iodine.

In children, I do not think too much stress can be laid on the great value of diaphoresis in the incipient stages. Warm baths, hot flannels, hot poultices, with warm inhalations, if practicable, and warm rooms, are admirable therapeutic agents. I have seen great relief from a bold use of ammon. acet. and nitre, with hot teas. For incessant cough, without much fever, I have seen inhalations of chloroform, repeated 4 to 5 times a day, give complete relief, and this, in one case, a child five months old. I am almost certain (many of our most prominent Baltimore physicians to the contrary, notwithstanding,) that I have procured excellent results by: ℞. Calomel, gr. $\frac{1}{8}$; tartar emetic, gr. $\frac{5}{16}\frac{1}{10}$; potass. nit., gr. $\frac{1}{4}$j, every 4 or 6 hours, for two or three days. If, after the acute attack, the cough still continues troublesome, and secretion tenacious, I have found potass. bromid., potass. bicarb., vin. ipecac., and syr. senegæ or scillæ to give favorable results; and also hydrate of chloral, with potass. brom., if carefully watched. In using any narcotic, in severe cases, great care is necessary to avoid narcotism, if the secretion be at all free.

The dyspnœa, which is a marked feature of the disease, may be produced by two causes: 1. From spasm of the bronchial muscular fibre. Relieved by chloroform, chloral, ether, morphia or opium—some form of narcotic.

2. From occlusion of tubes and filling of air cells, by excessive secretion; diagnosed by rales, etc. Assist expulsion by emetics boldly used, with stimulants and supporting treatment in the intervals. Supporting strength by beef tea, milk, cream, brandy, wine, etc., in small quantities, often repeated; but avoid full meals and prolonged sleep, as the one, by reflex action, may iuduce spasm and much coughing, and the other permits great accumulation of secretion, and hastens asphysia.

As fever falls, give bark, quininn, iron, ettc. I often prescribe the citrate of quinine and iron dissolved in good sherry wine, and have been much pleased with it.—*Dr. Noel.*

The Treatment of Cystitis.—Dr. A. W. Rogers writes; I have met with many chronic cases and very many cases of dysuria, which are commonly regarded as cases of irritation of the neck of the bladder. But, no doubt many of the latter are true cases of a degree of cystitis. Many more of the latter occur in females than in males. Some of them have been accompanied with blood in the urine, which, from the attending symptoms I had reason to believe came from the bladder. All the cases I have had of an acute kind have been relieved, and the most of them got entirely well under the use of the following means:—Rest, warm fomentations to hypergastric, pubic and perineal regions, and sometimes the moderately warm or tepid bath, warm demulcent drinks, small doses of spts. nitre dulcis and tinc. opii. camph. equal parts, say ʒss. of each other every hour; or pills of sulph. morph. grs. $\frac{1}{20}$ to $\frac{1}{10}$; or ext. hyoscyam. gr. $\frac{1}{2}$ to 1, one every two hours; or pills of camph gr. $\frac{1}{2}$, ext. hyoscyam. gr. 1, one every two hours. The mixture of equal parts of spts. nit. dulc. and tinc. opii. camph. frequently given in small doses will relieve a large majority of the acute cases of dysuria or mild cystitis, for which the physician is called to prescribe, such as occur from cold, some temporary menstrual or sexual trouble, and will temporarily benefit all of them. Where this remedy does not answer, the morphia with camphor, or the hyoscyamus and camphor will often succeed.

For all bleedings from the urinary organs gallic acid has always proved a reliable remedy, one which usually acts very promptly. It has so often succeeded in my hands that I have not looked for another. But I have no doubt that ergot is also an efficient remedy, and it is a matter of congratulation that its use in all kinds of hemorrhages is being proved to be so effective.

The beneficial effects of ergot in paralysis of the bladder following retention of the urine I have proved in several cases. The judicous use of this remedy will often save the physician and the patient much inconvenience. By its aid the use of the catheter may much sooner be dispensed with.

New Antidysenteric.—In the Vienna Exhibition are shown specimens of an "antidysenteric extract" prepared from the pods of the mangosteen (Garcinia mangostana,) a well-known plant in the East Indies. The extract has been prepared by Herr Gruppe of Manilla, and has been used in a large number of cases in hospital and private practice. It is said to be a trustworthy remedy in dysentery and in chronic diarrhœa, as well as in catarrhal disorders of the uterus, bladder and urethra, and generally in diseases for which astringents are indicated. It may be given in pills, or, especially to children, in the form of syrup.

Rules for Feeding Babies.—The following excellent rules, on the feeding of babies in general, are extracted from an essay recently read by Prof. A. Jacobi, M.D., of this city, before the Public Health Association. The rules in question were prepared especially as a guide to the public, and coming from such a source, are more than ordinarily valuable. We wish they could be placed in the hands of every nurse in the land. Embodying as they do the results of the experience of one of our highest authorities on the subject, they are also of particular value to the general medical practitioner. They are as follows:—

I. *Nursing Babies.*—Overfeeding does more harm than anything else. Nurse a baby of a month or two every two or three hours. Nurse a baby of six months and over, five times in twenty-four hours, and no more. When a baby gets thirsty in the meantime, give it a drink of water. *No sugar.* In hot weather—but in the hottest days only—mix a few drops of whisky with either water or food, the whisky not to exceed a teaspoonful in 24 hours.

II. *Feeding Babies.*—Boil a teaspoonful of powdered barley (grind it in a coffee grinder) and a gill of water, with a little salt, for fifteen minutes; strain and mix it with half as much boiled milk, and a lump of white sugar. Give it lukewarm, through a nursing bottle. Keep bottle and mouth-piece in a bowl of water when not in use. Babies of five and six months, half barley-water and half boiled milk, with salt and white sugar. Older babies more milk in proportion. When babies are very costive, use oatmeal instead of barley. Cook and strain. When your breast-milk is half enough, change off between breast-milk and food. In hot summer weather try the food with a small strip of blue litmus-paper. If the blue paper turns red, either make a fresh mess or add a small pinch of baking soda to the food. Infants of six months may have beef-tea or beef-soup once a day, by itself, or mixed with other food. Babies of ten or twelve months may have a crust of bread and a piece of rare beef to suck. No child under two years ought to eat at your table. Give no candies, in fact nothing that is not contained in these rules without a doctor's order.

III. *Summer Complaint.*—It comes from over feeding and hot and foul air; never from teething. Keep doors and windows open; wash your children with cold water at least twice a day, and oftener in the very hot season. When babies vomit and purge, give nothing to eat or drink for four or six hours, but all the fresh air you can. After that time you give a few drops of whisky in a teasponful of ice-water every ten minutes, but not more until the doctor comes. When there is vomiting and purging, give no milk. Give no laudanum, no paregoric, no soothing syrup, no teas.—*N. Y. Medical Record.*

Uses of Labarraque's Solution.—Dr. Robert T. Cooper, of Southampton, says, in the Dublin *Journal of Medical Science:*— Chlorinated soda has long enjoyed a favorable reputation as an application for suppurating and cancerous ulcerations, leucorrhœal and other catarrhal discharges, but I am not aware that it is generally supposed that, taken internally, it has a plainly marked stimulating effect upon relaxed states of the uterine and peri-uterine tissues; that it gives tone to the weakened uterosacral ligaments, inincreases vaginal contraction, removes the bearing down and tendency to prolapse, diminishes congestion of the very neck of the womb, thereby lessening very considerably leucorrhœal* and menorrhagic discharges; and yet this is what we claim for it, and more than this, that along with the removal of the original affection, the casual congestion comes, agreeably to the *tolle causam* principle, the more or less entire subsidence of sympathetic disturbances, the sacral, ovarian, rectal and vesical distress, the hypochondrial tumefaction, the gastric and precordial sinkings, the submammary stitch, chest pains, and headaches, a pretentious assertion certainly, but one we have verified. The method of administration we recommend is to put two or three drops of the ordinary commercial solution in half a tumblerful of water, and direct a dessert-spoonful of this tb oe taken occasionally. This will be found quite sufficient, and in susceptible persons disturbing effects even may be noticed. Should symptoms of the medicine disagreeing occur, such as rapid emaciation and influenza-like debility, which is one of its most uniformly unfavorable symptoms, it ought to be forthwith discontinued, and the patient advised to remain without medicine for some time.

*It does not, nor does any internal remedy that we know of cure a chronic leucorrhæa.

Oleate of Mercury in Treatment of Ringworm.—Dr. Kane (*London Lancet*) describes several cases in which he had used the oleate of mercury in treating ringworm. He says that over other remedies it has the following advantages. (1) It is a *certain remedy* if carefully applied. (2) It *produces no staining* or injury of the skin. This is of great importance where the disease appears in the face. (3) It is *painless* in its application, which is not true of the strong parasiticides. (4) It *readily penetrates* the sebaceous glands, hair-follicles, and even into hairs themselves. Thus it is more likely to destroy the fungus than spirituous or aqueous solutions of mercury.

In very sensitive skins the irritation sometimes produced by it may be avoided by reducing the strength of the solution, and applying it with a camel's-hair brush. The ordinary strength of the oleate is ten per cent.

Iodoform in Venereal Diseases.—Dr. Damear says: 1. That iodoform is a therapeutic agent, producing, more certainly and more promptly than all the others ordinarily employed, the cicatrization of ulcerative syphilids in general, under whatever form they present themselves.

2. That in the treatment of soft cancers, iodoform is in some sort a specific, by the promptness with which it produces cicatrization without pain.

3. That, in the treatment of simple or virulent buboes (non-syphilitic,) iodoform can be employed in the form of an ointment, as a resolvent, during the early stage, with more success than the blister and tincture of iodine; during the period which succeeds to the opening of the bubo, no other medicament can be compared with it for the rapidity with which it brings about the cure.

4. In all the preceding cases, when the suppuration is abundant, it is preferable to commence the treatment by the solution of iodoform in glycerine and alcohol; iodoform in powder ought to be employed in the second place.

5. Iodoform acts not only as a topical agent, but still farther, as a local anæsthetic. The rapid cicatrization which takes place is due, first, to the simplicity of dressing, which does not irritate the diseased parts; second, to the absorption of the secretions by the idoform powder; third, to the antiseptic properties of the iodoform powder; third, to the antiseptic properties of the medicament—above all, when it is dissolved in alcohol and glycerine; fourth, to the presence of iodine, which acts favorably on all veneral ulcerations in general.

6. Iodoform appears to us to be completely incapable of arresting the progress of phagedenism.

7. The employment of iodoform in cases of syphilitic affections should never dispense with internal treatment.

Treatment of Sloughy Ulcers of the Vulva.—Dr. Parrot has derived much benefit in the above cases from the employment of a concentrated solution of chlorate of potash, or cauterizations with nitrate of silver; but a means of treatment which has been most extraordinarily successful in his hands is the application of iodoform. Generally, in two or three days it stops the extending course of the disease, and cicatrization rapidly takes place. Iodoform is as successful in these cases as in bubo or fungus sores. It must be applied plentifully. The sores must be first well washed and cleansed, and then covered entirely with the powder. Sometimes, when the ulcer is very moist, and there is much loss of substance, it is useful to put on two dressings daily.—*La France Medicale.*

Chromic Acid.—M. Isambert extols the employment of chromic acid in scorbutic affections of the gums, ulcerations of the mouth, soft palate and pharynx; he uses it in solution more or less diluted. He has obtained very good results from its local application in laryngeal phthisis, and in syphilitic and scrofulous ulcerations of the parts above named, as well as for all the secondary manifestations of syphilis in these parts, as mucous patches, nodes, etc. Using the laryngoscopic, he has applied it by means of laryngoscopic sponges to epithelial vegetations and small warts appearing in the inter-arytenoid commissure, and in the vicinity of the cords. This severe cauterization should be applied only once in eight days. One of the best results obtained by application of this acid is the rapid repression of œdema of the glottis; it very rapidly shrivels up the tissues, considerably reduces their volume, causes the suffocation to cease after a short access of asthma, and thus renders tracheotomy unnecessary: the cauterization may require to be repeated. M. Isambert commences with rather strong solutions, and has in some cases used them in the proportion of fifteen grains of chromic acid to two fluid drachms of water, and, in a few rare cases, fifteen grains to one fluid drachms. Great circumspection is required in applying these more powerful solutions.—*Bull de therop.*—*Bouchardat.*

Cholera Infantum.—Dr. A. McBride, says: To a child one year old, with cholera infantum, give half a drop of fluid extract of belladonna, and you will see the pallid surface change to red in thirty minutes, and the flux will be checked; then give arsenic and opium (arterial and capillary tonics) to maintain the status of the circulation, and your case is quiet and comfortable. Add strychnine to give tone to the muscular tunic of the intestines, and there is only one thing lacking to complete the cure, and that is a diuretic: this may be nitrous ether and cubeb, or some other diuretic, Of course, all these articles are not necessary in every case.

If a case is in a very sunken or collapsed condition, I do not hesitate to give 20 drops tr. cantharides to begin with, or apply to the abdomen five inches square—twenty-five superficial inches—of fly plaster. The plaster should not remain on more than an hour or an hour and a half. The dose of 20 drops of the tincture will seldom require to be given more than once. Although this might seem a large dose for a child a year old, it is no more than equal to 120 minims for an adult—a dose which I give many times every year in similar conditions, congestions, stasis, etc.

The formula which I have used with unvarying success this season, in cholera infantum and all the choleraic diseases of adults, is the following: ℞. Sol. strychnia (1 gr. to the ℥). m. lxx.; sol. potass. arsenit; belladonna, fl. ext., aa. m. xxj.; spts. æth. nitrosi, m. cxl.; tr. cubeb, m. ccxxviij.; morphia, Munn's, grs. j$\frac{1}{6}$. M. Dose for a child one year old, 6 to 8 drops; for an adult, in a severe case of cholera or cholera morbus, 70 minims, or a teaspoonful. To be repeated once in three hours, more or less, according to to severity.

The first dose generally checks the symptoms decidedly, so that frequent repetitions are seldom necessary. Although the dose of belladonna, in the child's dose, is but little more than one-fourth of a minim, it very often brings out the peculiar red flush. I never made a combination of remedies in which the different ingredients so harmonized, and seemed so much to enhance each other's value. It may appear to some that the spts. nitre and tr. cubeb are added for some whimsical reason, but not so. It has long been a maxim with me, that when powerful or poison medicines were used, which did not, as part of their therapeutic effect, increase the quantity of some natural excretion, a diuretic should be used, to carry out of the system the results of their disintegrating effects; and I find, in the use of arsenic especially, this practice has an excellent effect, and so I think of the others.

Pumpkin Seed for Tape-worm.—Prof. N. S. Davis, (*Chicago Medical Examiner*,) says: During the last five years I have caused the expulsion of more tape-worms by the use of pumpkin-seeds than by any other remedies. Success in the use of this remedy, however, depends almost wholly on the *manner* of its use. Two ounces of the seeds should be thoroughly bruised or broken up and put into a pint of water boiling hot, in the evening. Stir them up several times while cooling, and let the dish stand until morning. Let the patient retire at night without any supper, and in the morning drink the whole pint of pumpkin-seed infusion, instead of breakfast. If the bowels do not move freely by one or two o'clock, p. m., let him take a tablespoonful of castor oil, mixed with a teaspoonful of oil of turpentine. After it has operated freely he may take food as usual. I have known this method to succeed in expelling the whole mass of the worm in a goodly number of cases.

Syrup of Borax.—℞. Borax, ʒiv.; syrup simp., ℥x. Dissolve by the aid of heat. For laryngeal catarrh, a tablespoonful to be taken from 7 to ten times a day, and swallowed without dilution, and avoiding to drink immediately after, so as to prolong the contact of the remedy. E. S. W.—*Cincinnati Lancet and Observer, September*, 1873.

Phosphorus in Neuralgia.—Dr. Slade-King, (*Medical Times and Gazette,*) speaks highly of this drug in neuralgia, particularly in tic-douloureux and hemicrania. He says: The dose of phosphorus should not exceed one-twentieth nor be less than one-thirtieth of a grain, to be repeated till an entire grain is taken in all. If the pain is not then greatly relieved or cured, it is of no use to push the administration of the drug further. The absorption of phosphorus into the blood is rapid, and its effects transitory in the extreme. Its best mode of administration in neuralgia, therefore, is in minute but frequent doses, commencing with the one-twentieth or one-twenty-fifth of a grain every two hours. The patient must partake of a little mucilaginous or farinaceous nourishment some ten minutes previous to each dose. When eight doses have been taken in this manner, increase the interval between each dose to four hours, and finally to eight hours. Used in this way I have found it most successful in its results, and have never noticed any unpleasant effects. Its action for good has been more rapid than any drug which was not narcotic or anodyne, and its results lasting in most cases. The administration of phosphorus in the solid form is neither convenient in dispensing nor safe to the patient; its solution in ether is open to the objection of the smell, which cannot be avoided, and to its easy precipitation, which defect may, however, be easily met by adding it to equal parts of mucilage and syrup. It is in this form that I am compelled to prescribe it, not having been fortunate enough to procure the solution in superheated oil of sweet almonds, recommended by Méhu, which, when enclosed in capsules, would be probably the most convenient formula.

Ulcerations in Chronic Diarrhœa.—Dr. Theo. Mead (*N. Y. Medical Journal*) writes: My treatment is, to inject into the bowel half a drachm of chlorate of potash rubbed up in half an ounce of glycerine, and mixed with three to four ounces of warm water, two or three times a day, the patient to be confined to the bed, with instructions to hold the enema as long as possible. The first injection will not be held over half a minute, and not that length of time if the rectum be much affected. But in a few days the trouble in this respect will be greatly overcome, and, as the ulcerations heal, greater tolerance of the medicine will be evinced. After these injections have been used for from seven to ten days, the whitish membranes and *debris* of the ulcers, provided it be an old case, will be passed with the stools, looking like scraped lint. This occurred in the first case I had, to the no small surprise of my patient. The general health of the patient, of course, needed continued attention, and such medication as the case indicated was prescribed.

VALUE OF DRY-CUPPING.—Dr. B. H. Washington, (*Nashville Medical and Surgical Journal,*) says:—I have cured a case of tetter of thirty years' duration, for which forty doctors had prescribed in vain, by dry-cupping alone, and another case of twenty years' duration. I have often used it in tedious labors to bring on uterine pains. Professor Fenner reported a labor case of his own, in which the woman was in labor thirty hours, and he had decided that it was necessary to use instruments, when he recollected having read my article recommending dry-cupping in tedious labors; he tried it, and the woman was safely delivered in thirty minutes. I have myself sometimes relieved a patient in fifteen minutes from the time the cups were put on.

I have completely taken off muscular resistance in dislocation of the humerus, by dry-cupping, and replaced the bone so easily, that the patient scarcely experienced any pain at all. I have cured sick headache completely by it. I have cut short the sympathetic disturbances of pregnancy, and, by keeping the irritation at home, in the uterus, enabled the patient to enjoy fine health, while in previous pregnancies she was miserable. I have cured a case of total paralysis in the arm, from a gun-shot wound, and no other remedy would have caused the absorbents to take up the interstitial deposits, and allow nervous communication to be renewed. I have stopped, immediately, a case of puerperal convulsions. And as an excito-secretory remedial agent, I have never been able to find anything to compare with it.

RESINA COPAIBÆ.—A notice of the use of the resina copaibæ in Dr. Wilk's wards at Guy's Hospital as a substitute for the simple copabia, where a diuretic was required, has caused numerous inquiries as to the best mode of its administration. Dr. Wilks states (*Lancet,* June 21, 1873) that Mr. Gerrard, the dispenser at the hospital, advises the following formula: "Resin of copaiba, three drachms; rectified spirit, five drachms; spirit of chloroform, one drachm; mucilage of acacia. two ounces; water to make twelve ounces." An ounce (containing fifteen grains) to be taken three times a day.

Dr. Wilks takes this opportunity of saying that he has received numerous communications acknowledging the superiority of the pure resin over the oleoresin in the absence of odour, at the same time that the diuretic property remains. One patient, a medical man suffering from cardiac dropsy, and who had taken the ordinary remedies without much benefit, commenced the resin in the form of a pill, taking three pills, containing five grains of the resin in each, three times daily. Diuresis at once commenced, with a departure of all the fluid from the abdomen and legs.

Remedy for Dysentery or Flux.—Dr. Thos. G. Gooch, (*Nashville Medical and Surgical Journal*), Says: I have concluded to submit to your consideration a remedy that I have been using for several years, for dysentery or flux, and which, in my hands, has given the most prompt and certain relief, and ultimate cure, of any and all other remedies I have ever known to be used in the treatment of that painful, and, in many cases, fatal disease.

The drug mostly relied upon was the sulphate of zinc. I had been using a preparation of the sulphate of zinc ever since the year 1821, in all cases of inflammation, sloughing sores, burns, sore eyes, etc., with astonishing good effect, and I determined, if I had a case of flux, that I would try it, by applying it to the lower bowel by injection.

*How to prepare the solution of Zinc.*—Take half an ounce of the sulphate of zinc, burn it on an iron plate (a fire-shovel will do, if well cleaned,) until all the water of crystalization is dried out. It will adhere very closely to the iron, but must be scraped off. Pulverize well, and mix it with the yelk of one egg, boiled or roasted until tolerably hard. Put the mixture into one quart of boiling water, and heat the end of a common bar of steel to a bright red heat, and stir the bar in the water as long as the heat of the steel will continue to boil the water. Settle, strain, and bottle. Half an ounce, the dose, by injection, to be repeated in 6 or 8 hours.

Bismuth in Skin Diseases.—A corrispondent writes:—"The value of bismuth in alleviating the intense itching and irritation which accompanies chronic eczema and other forms of skin diseases is less known than it ought to be. In some obstinate cases, in which the inflamed condition of the derma is aggravated and kept up by the constant scratching and rubbing which the patient finds it impossible not to indulge in, I have found an ointment containing bismuth in the proportion of half a drachm of the subnitrate to the ounce of simple ointment, rubbed up with a little spirits of wine, and applied freely to the skin, give great relief. I was indebted to a work by Dr. McCall Anderson for the suggestion, and have certainly found the ointment far more efficacious as a soothing remedy than the benzoated zinc ointment more commonly used. Dr. McCall Anderson, in recommending bismuth, observes that the ointment must not be made with benzoated lard, or the reverse of a soothing effect may be produced."—*Medical Times and Gazette.*

Treatment of Croup.—Dr. Welch recommends the use of iodine, given internally, in the treatment of croup, and relates, in confirmation, a case treated successfully by doses of one or two drops of the tincture every half hour.—*The Doctor.*

The Best Form for Administering the Phosphates.—The great value of the phosphates as therapeutic agents in certain kinds of diseases has led Coirre to inquire into the best way of employing them, and especially one of the most valuable, the phosphate of lime. In order that this remedy may be readily taken up by the system, he recommends that it be given in doses of 10 grs. dissolved in ℥ss. of water, to which a few drops of hydrochloric acid has been added. This latter represents the free acid of the gastric juice. It aids in the absorption of the phosphate, and is of itself useful, as in small doses it promotes digestion. Its use is indicated in diseases which cause disturbance of the digestive and nervous system, such as chronic purulent discharges, chlorosis, tuberculosis (where calcification of tubercles may be desirable,) and similar affections, in the osteomalacia of pregnant and nursing women, where the phosphate of lime is collected in the blood of the mother for the benefit of the child, but to her own serious detriment.—*Press Med., Schmidt's Jahrb.*, 4, 1873.

Quinine by the Rectum.—Sulphate of quinia is one of those agents which produce their proper effect through the rectum, particularly in children, in nearly the same quantity as when taken into the stomach. The *Detroit Review* remarks on this subject: "In our experience, quinine will do its work just as well when mixed with cocoa butter and pushed into the rectum, as when taken by the mouth. This can be done with less trouble and discomfort than attends the swallowing of sugar-coated pills. The doctor, patients and attendants who have tried this method of giving quinine to children and fastidious people, will be sure to remember and practice it in similar cases. There is no way equal to this for administering narcotics when the stomach is weak or the patient unwilling to take the medicine per mouth."

Treatment of Biliary Calculus by Choleate of Soda.—Schiff admits that these calculi are formed of cholesterin, not because this substance is formed in too great abundance, but because the bile does not contain the principles which maintain it in solution. These are the cholates and choleates of soda and potassa, more than the alkalinity of the bile which dissolves the cholesterin. Schiff therefore advises the administration of eight grains of choleate of soda, to be given twice daily, and increased until "saturation" is indicated by irregularity of the pulse, which becomes slower during repose, and accelerated by the least effort. The dose may then be diminished, but not entirely suspended—a considerable time, a week at least, being required for the remedy to produce amelioration of the symptoms.—*Imparziale and Gaz. Heb.*

Cod-Liver Oil Mixture.—A preparation that has met with much favor, under the above name, has been made by the writer from a formula given him by Mr. Hassard, of Philadelphia. It is made as follows: ℞. Fresh eggs, No. iv.; lemon juice, q. s. Place the eggs in a suitable vessel, and pour over them sufficient lemon juice to cover them, and let the whole remain for 24 to 48 hours. Then pass the whole through a strainer, and add, with agitation, the following, in the order given: To the lemon juice and eggs add an equal volume of honey, cod-liver oil, and brandy or whisky. The whole forms a permanent emulsion, and will keep good during the summer months for a month, and longer in cooler weather. The taste of the oil can be covered by the addition of a few drops of oil of winter-green, or oil of bitter almonds. This mixture is pleasant to take, and a valuable therapeutic agent.

P. S. Glycerine may be substituted for the honey. E. S. W.—*Cincinnati Lancet and Observer*, September, 1873.

Glycerin of Borax in Facial Erysipelas.—Prof. D. M. Salazar, of the Hospital Nacional, Madrid, reports that he has cured eight cases of facial erysipelas in forty-eight hours by his remedy. Notwithstanding the rapidity with which the affection disappeared, there were no consecutive pathological affections. In one case the disease had existed three days before treatment was commenced, and there were bilious vomiting, intense cephalalgia, high fever, inflamation of the entire face, and some phlyctenulæ in the vicinity of the right lower eyelid and the root of the nose. He applid the solution to the diseased parts with a brush, and then covered them with a mask of raw cotton. After twenty-four hours all the symptoms, local and general, were notably diminished, and the next day all the phlyctenulæ had disappeared and desquamation was commencing.—*El Amflt. Anat. Españ., March,* 1873.

Remedy for the Rapid Cure of Catarrh.—Hamilton was cured in about ten hours of a very severe catarrh, by the following mixture: Acid carbol. gtt. 10, Tinct. Iodi, Chloroform aa 7. 5 gramme. Some drops were put into a test-tube, heated over a spirit-lamp, and when evaporation commenced held under the nose. After two minutes this procedure was repeated, which was followed by sneezing several times, when the troublesome symptoms soon disappeared.—*Neues Fahrb. fuer Pharm.*

Pruritus Vulvæ.—Take of bichloride of mercury, one part: alum, twenty parts; starch, one hundred parts; water, twenty-five hundred parts. Mix. S. Apply freely to the part.—*Revue de Therap.*

Morphine and Cimicifuga Racemosa in Puerperal Convulsions.—Dr. C. R. Gilbert, Metamora, Fulton county, Ohio, communicates to us his experience with morphine and cimicifuga in puerperal convulsions, which experience is so gratifying as at once to command itself to the profession.

He states that with these remedies he seldom, if ever, fails to control the convulsions whenever the system is placed under their influence, and expresses himself confident by reason of practical observations extended through several years, that ordinarily this course of treatment will prove efficient.

He gives the morphine and fluid extract of cimicifuga alternately, both in large quantities, and repeated *pro re nata* until the system be manifestly brought under their influence, and succeeds in arresting the convulsions in from one to two hours.—*Journal Materia Medica.*

Opacities of the Cornea.—M. Ansiaux, Professor of Clinical Surgery in the University of Liege, employed, with success, the following: ℞—Cadmii sulplat, gr. j.; mucilag. gum-acaciæ, tr. opii (Sydenham) aa. ℥ij. M. et. ft. collyr. The dose of cadmium should be increased, if it is well tolerated, and may be carried even to twelve grains, though this is rarely necessary, in the author's experience. The collyrium should be applied, by means of a small brush, two or three times a day, and the patient directed to close the eyes for ten minutes after its application, in order that the lotion may not be carried off by the tears. Laudanum having been proposed for the cure of these opacities, one might attribute the numerous cures of Prof. Ansiaux to its use in the prescription, but the elclusive use of laudanum has not been followed by the same success.—*La France Medicale*

Domestic Pepsin.—Dr. N. L. Fulsom (*Am. Jour. Pharmacy*) writes: I see, in the Journal of May 22d, an article on pepsin, by Dr. Hoskins, of Lowell. I think his remarks will do good.

I am using what I call *domestic* pepsin, consisting of the inside of the gizzards of chickens, turkeys, ducks or geese, or the stomachs of calves or little pigs. Dry them on a stove in a plate, and then bruise them, and give a third of a teaspoonful of the powder in syrup a few minutes before eating. Some of the country people dry the gizzard itself and then grate it and give that powder the same way for dyspepsia.

I think this crude, inelegant *domestic* pepsin far superior to pepsin made from *macerated* pigs' stomachs, and it costs the poor patient next to nothing. I direct the patient to obtain and dry these skins and bruise them.

Page(s) missing

Abortive Treatment of Boils.—The *Cincinnati Lancet and Observer* has a note from Dr. C. B. Hall, stating that the following, applied to boils, with a camel-hair pencil or feather, gives great relief in a very short time. The inflamed surface, and a little beyond all around, should be painted with the medicine every fifteen minutes, or as fast as it dries, till a good thick coating covers the part. The throbbing, tensive pain, and the intense tenderness will be promptly relieved; the redness will subside; the smooth, shining integument will shrink and become wrinkled, and comfort will succeed torment. If the boil is in the first stage, it will disappear without sloughing. If slough has already formed, it will be quickly separated, and the cure soon complete: ℞. Tr. arnica flowers, ʒi.; tannic acid, ʒss.; gum acacia pulv., ʒss. M. It should be used as soon as prepared.

On Aromatic Tincture of Assafœtida.—Mr. L. Myers Conner says, in the *American Journal of Pharmacy*, this tincture has such an unpleasant smell and nauseating taste, that it cannot be given in every case required. Frequent requests of physicians to prepare a tincture that would be more pleasant to the taste, and produce the same effect without the addition of water, have induced him to make some experiments. The formula offered has been tried, the aromatics being no objection, either in properties or preparation; it can be made at any time, also keeps well. ℞. Tinct. assafœtida, U. S. P., ℥viij.; tinct. orange-peel, U. S. P., ℥ij.; ess. peppermint, ℥iij. M. Dose, one and a half to two fluid-drachms, without the addition of water.

Chloral as an Application in Fetid Ulcers.—MM. Dujardin-Beaumetz and Hirne communicated at a recent meeting of the Paris Medical Hospital Society the result of the investigations they have been making into the "anti-putrid and anti-fermentiscible properties of solutions of hydrate of chloral and their therapeutical applications." Their attention was first drawn to the subject by the remarkable success which attended the application of a solution of chloral diluted by 100 parts of water to a vast eschar of the buttocks which occurred during an attack of typhoid. Since then they have employed the solution in the treatment of various wounds in bad condition, and in suppuration occurring in closed cavities.

Glycerite of Lime used in Burns is said by De Breyne, to soothe the pain and to prevent inflammation or diminish its intensity; it is prepared from recently slacked lime, one part; glycerin, fifty parts; chlorinated hydrochloric ether, one part.—*L'Union Pharmac.* 1873, *June.*

GAS IN STOMACH—Dr. F. Sibson (*British Medical Journal*) says: This gas is secreted, not by the structure of the stomach, but by the food. If you cut off the supply of food, sooner or later, when that already in the stomach has passed away, relief will be obtained. But we cannot cut off the supply of food; therefore we must select such kinds of food as give the greatest amount of nourishment, and tend to set free the least amount of gas; for although every kind of food will produce gas when the powers of digestion are weak, yet some kinds will do it much more freely than others. My rule is simply this: Take no bread, but eat biscuits instead. Take no solid vegetables, but use lemon juice freely. Squeeze some of it over all your food. Shun soups, cucumbers, and fruit. Prefer meat, fish, fowl, or game, tender, but perfectly fresh, lightly cooked, and juicy—not hard and dry; or take it thoroughly soft and fragrant, as sent up by the hand of the artist. Take no rich sauces, or, indeed, any other sauce, than wholesome, fresh lemon juice or mushroom ketchup; no twice cooked, or salted, or seasoned, or dried meat. Let everything be fresh and savoury. Under this simple rule of substituting biscuit for bread and fresh lemon juice for vegetables, relief will often be obtained from distressing symptoms indicating danger. Let the food be agreeable to the eye, fragrant to the nostril, and savoury to the palate; and, invited by these senses, the influences of the nervous system will bring those juices into the mouth and stomach that are required for the digestion of the food—juices that are attracted if the food is attractive, repelled if the food is repulsive.

QUININE PILL MASS.—M. Berquier, of Provins in the *Repertoire de Pharmacie*, suggests the following formula for a quinine pill mass:—℞. Sulphate of quinine, 30 grains; Powdered gum, 5 grains; Glycerine, 10 grains. Mix the gum with the glycerine and then incorporate the quinine, beating it well in a morter.

This is said to give a mass of good pilular consistence, which retains its softness, and can be easily rolled into pills. It can readily be worked up with other ingredents, and is not bulky. Three grains of this mass are equal to two grains of sulphate of quinine.—*Medical and Surgical Reporter.*

THE TREATMENT OF BURNS AND SCALDS.—Dr. De Breyne highly recommends the following treatment in *L'Union Pharmaceutique:* Hydrate of lime (newly precipitated,) forty-five grains; glycerine, five ounces; chloric ether, forty-five drops. It makes up a transparent, colorless liquid, with an agreeable odor, and an alkaline reaction, according to the amount of hydrate of lime. It calms the pain and prevents or abates inflamation.

Epithelioma.—Dr. M. Marsh, (*Medical and Surgical Reporter*) writes of Prof. Mussey's method of treatment, remarks: When the disease was in its warty, scaling, oozing stage, he prescribed with success nitrate of lead in solution, applied with a sponge three or four times a day, and in his opinion often effecting a perfect cure. When ulcerated and indurated to considerable extent he used the knife, and fearlessly used it, especially when the destructive, ulcerative character was unmistakably manifested. According to my recollection the microscope was not applied then to the detection of cancer cells, or at least he did not so apply it.

I will give one case of many to illustrate Dr. Mussey's treatment. Three years ago I was applied to by a married lady, æt. 25, primipara, with a lump or pimple on her nose, covered by a scab of three years' standing, resisting all remedies that had been used. It itched at times intolerably, with a sticking, pricking pain, scabs falling off and reappearing, leaving a red surface. I prescribed the old Dr. Mussey remedy:—℞. Plumbi nitras, ʒij.; aquæ rosar., ℥iv. M. Applied three times a day.

In four weeks her nose was smooth and cured, relieving her from wasting nervous excitement and continued anxiety. And thus she remains, in good health. A more perfect cure could not have been effected if I had cut her nose smooth to her face.

Introduction of the Stomach Pump Tube.—Dr. McEwen, in the *Glasgow Medical Journal, Feb.*, 1873, recommends that the head should be bent forward on the introduction of the tube, instead of backward, as is generally taught in books. When the head is thrown backward, he says, the spine becomes convex anteriorly. When the tube is passed along it has a tendency to impinge upon the larynx; but when the head is bent forward, the mouth, pharynx, and œsophagus form a curve along which the tube glides gently into the æsophagus and at the same time is directed away from the larynx.

Lime-water in Stings of Bees and Wasps.—M. Dauverne states (*L' Unin Med.*, October 25) as the result of numerous trials that the pain and suffering caused by these may be immediately assuaged by the application of lime-water—a remedy which may always be prepared at once by the aid of a little quicklime and a glass of water.—*Med. Times and Gaz.*, Nov. 1, 1873.

Wright's Cure for Headache following Alcohol Debauch: Take a solution of acetate of ammonia, tincture of bitter orange-peel, syrup of bitter orange-peel, each 20 parts, water 500 parts. To be given in repeated teaspoonful doses.

Treatment of Aged Persons.—Mr. Habershon, in a clinical lecture (*Guy's Hospital Gazette*, November 8) on the case of an old man who died suddenly after exposure to cold, remarked: "The old man died simply from the shock produced by coming out into the cold and fog, which, though only an inconvenience to us, was sufficient to lead to a fatal result in one whose circulation had become enfeebled, and whose vital force had so nearly lost its power. I am reminded (Dr. Habershon says) by this case of an instance of longevity communicated to me by a gentleman the other day: his mother had died at the age of one hundred and two, during the winter month, 'had refused to get up, saying that she was only warm in bed.' I have no doubt that it was owing to this uniform warm temperature that she lived so long; and I mention the instance as a recommendatfon to you, when you have to prescribe for old people, to advise that they be kept warm. You should also look carefully after their nourishment. Old people cannot eat large meals; therefore they must take them more frequent. Many old people will wake up about three or four o'clock in the morning. It is a good plan that they should have some nourishment then; otherwise, the interval between the night and morning meals is too long for their declining strenght. It is by care in such minutiæ, that we may prolong the life of the aged."—*Lond. Med. Record*, Nov. 26, 1873.

Lumbricoides.—Prof. Davis, says: They are generally easily expelled by the judicious uso of either oil of turpentine, santonine, spigelia marylandica, or carbolic acid, aided by mild laxatives. For convenience of exhibition, certainty of effect, and safety from bad consequences, the santonine is the best remedy for this species of worm. It may be given in doses of from one to five grains, according to the age of the patient, repeated every four hours, until three or four doses have been taken, and then follow it by a cathartic.

Belladonna in Intestinal Invagination and Hernia.—M. Gallicie (*La France Medicale*,) in a paper on belladonna, says it is the special medicament for intestinal invagination, strangulated or not, as also for inflammatory elements. In both cases, however applied, its first effect is to alleviate the intensity of pain, and to diminish and arrest the vomiting.

Gout Pills.—Dr. Theo. H. Jewett, of South Berwick, Maine, recommends the following formula of pill as very efficacious in rheumatism and gout. ℞. Colch. ex. acet.; Calomel; aloes soc.; ipecac, aa. ʒi. M. ft. mass. divid. in pill, lx.

Depilatory.—Prof. Bœttger recommends the following as safee 1 part of crystallized sulphhydrate of sodium is rubbed to a very fine powder, and mixed with three parts of prepared chalk. The mixture keeps well in closed vials. Mixed with water and applied to the skin, the hair becomes soft in two or three minutes and is readily removed by water.

[This appears to be an improvement on Boudet's depilatory, which consists of 3 parts of crystallized sulphhydrate of sodium, 10 parts of quick-lime and 10 parts of starch.—Editor.]—*Am. Jour. Pharmacy.*

Digitalis an Anaphrodisiac.—M. Gourvat, in the course of a paper published in the *Gazette Med. de Paris* on the action of digitalis says: "When digitalis or digitalin is administered for some time to a man in full possession of sexual power, these become gradually weakened, the propensities disappear, formation of the liquor seminis diminishes, and may at last cease altogether. The anaphodisiac properties of the drug are the secret of its good effect in spermatorrhœa."—*American Practitioner.*

Iodoform in Diseases of the throat and Nares.—Dr. R. P. Lincoln, of New York, gives in the *Medical Record,* September 15, in detail, a number of cases of chronic nasal catarrh, and ulceration of the naso-pharyngeal space in which he used *powdered iodoform* with the most satisfactory results, in some of which the disease had resisted most of the usual applications. This remedy was also used in the form of an ointment, a *drachm to the one ounce of lard.*

Lotion of Acetic Acid for Baldness.—The following lotion is said to be superior for a shampooing liquid, for removing dandruff, and useful and pleasant application for baldness. It is, of course, moderately stimulating, and in those cases in which the hair-follicles are destroyed, but have become merely inactive, we should think it might prove both efficacious and agreeable: Take of acetic acid 1 drachm; cologne water 1 ounce; water, to make in all 6 ounce. Mix.—*Druggists' Circular.*

Chronic Catarrh of the Pharynx Treated by Galvano-Caustics.—In an article published in *Deutsche Zeitschrift fur Chirurgie,* Dr. Carl Michel, of Cologne, advocates the use of galvano-caustics in chronic catarrh of the pharynx. He mentions seventy cases in which he was entirely successful, and in many of which inhalations, cauterizations with nitrate of silver employment of mineral waters, etc., had failed.

How to Deprive Iodine of its Stain.—Add a few drops of phenic acid to the tincture, and it will not stain; moreover, the tincture is more efficacious, and its action more certain. M. Bogs recommends the following formula for use in injections: Alcholic tincture of iodine, three grm.; phenic acid, six drops; glycerine, thirty grm.; distilled water, one hundred and fifty grm. This preparation is superior to all others in the treatment of blenorrhagia and leucorrhœa.—*France Medicale.*

Psoriasis.—Dr. Buck, of Berlin, has found the external application of acetic acid the best of all remedies in psoriasis. He first softens the skin eruption by soap and water baths, and rubs off the epidermis scales with a soft brush. He then paints the spots affected with dilute acid, so long as the patient will bear it. This is done frequently. No scar is left, and the treatment requires four to six or eight weeks. The same application is useful in warts and icthyosis.

Carbolic Acid in Obstinate Leucorrhœa.—Dr. Whitaker, of Cincinnati (*Cincinnati Lancet and Observer,*) speaks highly of carbolic acid in the treatment of obstinate leucorrhœa. The application is made upon the cotton-wrapped sound. The instrument, so enveloped, is first introduced and the uterine surface cleansed; a new layer of cotton is substituted, medicated by immersion in a solution of proper strength, and applied to every portion of the uterine cavity.—*Medical Examiner.*

To Prevent Regors Following Catheterism.–Dr. Gouley says: Mr. Long speaks highly of two-minim doses of Fleming's tincture of aconite, for preventing rigors in cases where they had occurred after catheterism. This is, I believe, another excellent remedy, but I would not take it in exchange for quinine. I have lately, however, given it in combination with quinine.

Dried Meat for Medical Purposes is prepared by Dannecy of Bordeaux, by cutting fresh meat finely, spreading upon muslin, drying rapidly in a current of air and rubbing into a brown powder, which is almost inodorous, and has a slightly saline taste. It is readily taken by patients, spread upon bread, or a teaspoonful of it mixed with a cupful of broth or soup, or by children, if baked into biscuit.—*Ibid. from Bullet. Gener. de Therap.* lxxxii.

Oxide of Silver in Menorrhagia.—℞. Argent. oxid., grs. jss.; ext. tarax.; tragacanth pulv., aa. grs. v. M. f. pil. No. 6. One to be taken each day.—*The Cincinnati Medical News.*

IMPOTENCY.—Prof. D. Hayes Agnew, of Philadelphia (*Medical and Surgical Reporter,*) in a clinical lecture on impotency, after stating that the treatment must be largely moral, and urging the abandonment of onanism or excessive venery, says there is no better internal treatment than phosphorus and strychnia—$\frac{1}{50}$ grain of the former, and $\frac{1}{30}$ of the latter—made into a pill, and administered three times daily.—*Clinic.*

FACIAL PALSY.—Dr. Detmold says of an obstinate case:—"I determined to tray what mechanical means would do. I bent a wire into a hook, which I put into the drooping corner of the mouth, and, drawing it up, bent the wire over and behind the ear. I recommended the patient to keep it on overnight, trusting that, by entirely relaxing the paralyzed muscles, and supporting the dragging weight, I might somewhat relieve the defect." Prompt amelioration followed this method of treatment.

EAR-COUGH.—Dr. Solis Cohen, of Philadelphia, had a patient subject to ear-cough, a peculiar spasmodic cough produced by touching any part of the external auditory meatus. Gave him a dose of twenty grains of quinine, which induced a cough exactly similar to the ear-cough from external irritation. This cough continued until the influence on the system subsided.

SPOTS ON THE CORNEA.—A number of cases of this disfigurement were treated by Professor Anciaux, of Liege, "with wonderful success." His application is a weak solution of sulphate of cadmium in equal parts of mucilage of acacia and Sydenham's laudanum. With this the spot is painted two or three times a day. After the application the patient should close the eyes for a few minutes, that the solution be not washed away by the tears.

GONERRHEA TREATED BY CREOSOTE.—Dr. Huyette, of Phelps county, Mo., in the *St. Louis Medical Journal* for September, extoles the action of creosote in the treatment of gonorrhea. He gives from one to three drops in emulsion, three times a day and at late bed-time. He claims that it acts as a specific destroyer of the disease, and prefers the internal to the topical use.

NERVOUS OR SICK HEADACHE.—℞. Bomide Potassium, grs. xv.; Fluid Extrct Valerian, ʒ jss.; Tinc. Opii. Deodorized, gtt. xij. M. As a dose for neuralgia, nervous headache or pains of dysmenorrhœa, to be repeated every hour until relief follows.

[The addition of muriate of ammonia would make the mixture all the better.—EDS.]

Atropine is well known as a good remedy in all kinds of corneal affections. Carbolic acid should be combined with it. In this combination, we shall have the best known remedy for small pox keratitis.

The following is a good prescription. ℞. Atropine sulph. gr. iv.; acid carbolic, gr. v.; aquæ distilat, ℥j. Mix. To be dropped into the eye three to five times a day, according to severity of the keratitis; two or three times at night, if there is much pain.

Uterine Tonic.—Take fluid extract of hamamelis virginica, two ounces; fluid extract of helonias dioica, one ounce; fluid extract of euonymus atropurpureus, one ounce; pyro-phosphate of iron, two drachms; mix, and add to four pounds of simple syrup. In teaspoonful doses four or five times a day, this syrup will remove uterine congestion, and cure the whites or leucorrhœa. So says Dr. Paine.

Chlorine-water.—According to Mylius chlorine-water is best administered with syrupus simplex, by which it is also conserved better than with honey, glycerine, or syrupus althaæ. Mucilage of gum will also answer well. The author finds that chlorine-water without such addition will give off its chlorine much more rapidly.

A Palliative in Painful Urination.—In retention of urine from stricture, in hypertrophy of the prostrate, after operations on the urethra. etc., Dr. Cazenavi recommends the employment of ice suppositories. A piece of ice, of oval shape and the size of a chestnut is inserted in the rectum. The relief is prompt and most satisfactory to both patient and physician.

Laxatives.—A new remedy has been introduced as a laxative, which is said to be preferable to many of the salines, on account of its agreeable taste. It is the sulphovinate of soda in two drachm doses.

Another very efficient and much used laxative compound is the following: ℞. Ext. colocyth, co., gr. vi.; Ol. caryophyl, gtt. ij. M. Divide in pilulæ No. ij.

Glycerole for Chapping of the Skin.—℞. Oxide of zinc, gr. xx; Tannic acid, gr. xv; Glycerin, ʒix; Tincture of benzoin, ʒss; Camphor, gr. xv. M.—*Canada Medical Record*.

Enlarged Tonsils have been very successfully treated by hypodermic injections of tr. of iodine ($\frac{1}{6}$ to $\frac{1}{4}$ of a syringe full,

For Ulcers at the Rosevell Hospital.—The dressing which gives the most satisfactory results for cleansing a foul, sloughy, ulcerated surface, preparatory to strapping, consists of a solution of chloride of lime, one-half ounce to a pint of water, and applied three times a day upon a cloth or pledget of lint.—*Medical Record.*

Treatment of Inflamed Piles in the Puerperal Bed.—M. Joulin applies to a hemorrhoidal tumor a piece of ice contained in a caoutchouc sac. The sac should be enveloped in a wrapper of linen, and the ice must be carried well up the rectum to avoid too painful a reaction.—*Gaz. de Joulin.*—*Gaz. de Hop.*, Aug. 7, 1873.

Remedy for Chronic Hoarseness.—In chronic hoarseness arising from thickening of the vocal cords and adjacent membrane, the ammoniated tincture of guaiacum is often a very efficacious remedy. It may be appropriately mixed with equal parts of the syrup of senega, and a teaspoonful of the mixture, given two or three times a day.—*American Practitioner.*

Nervous Dyspepsias.—For nervous dyspepsia, attended with constipation, irregular apetite and insomnia, use as a laxative, the following pill: ℞. ext. hyoscrami: ext. scutellaria; sulph. ferri aa. gr. xx; pulv. alos; blue mass, aa. gr. xv. M. div. in pills, xx. One to be taken before each meal until the peristaltic action is established, and then one at bed time.

Sciatica.—The treatment by the actual cautery merits a more extended trial. It will be found especially useful in cases where the affection is due not so much to any rheumatic element in the system, as to local injury to the component fibres and funicular sheaths of the nerve itself.

Antidote to Carbolic Acid.—Dr. T. Hasemahn, from numerous careful experiments, both chemical and medicinal, advocates the use of a strong solution of saccharate of lime, of course to be taken as soon as possible, as an antidote to carbolic acid.—*Medical News.*

Hæmoptysis.—The editor of the *New Remedies* recommends the *atomized* solution of the liquor ferri subsulphatis U. S. P. as a rational treatment of hæmoptysis.

Hydrochlorate of Ammonia in Portal Dropsy.—Dr. Spencer Thompson, of London, recommends this in doses of ℈i. to ℥ss. every six or eight hours.

